# Assessment of lipid peroxidation and total antioxidant capacity in patients with breast cancer

**DOI:** 10.37349/etat.2025.1002284

**Published:** 2025-01-02

**Authors:** Abdullatif Taha Babakr, Mohamed Mahmoud Nour Eldein

**Affiliations:** ^1^Department of Medical Biochemistry, Faculty of Medicine, Umm Al-Qura University, Makkah 21955, Saudi Arabia; ^2^Faculty of Medicine, Ain Shams University, Cairo 11566, Egypt; Hospital del Mar Medical Research Institute, Spain; IRCCS Istituto Romagnolo per lo Studio dei Tumori (IRST) “Dino Amadori”, Italy

**Keywords:** Oxidative stress, breast cancer, antioxidants, oxidized LDL, free radicals, cancer biomarkers

## Abstract

**Aim:**

Breast cancer (BC), a disease in which abnormal breast cells grow out of control and form tumors, is a prevalent life-threatening disease worldwide. Oxidative stress has been implicated in the development and progression of various cancers, including BC. Assessing lipid peroxidation and overall antioxidant status in BC offers valuable information on disease progression, patient prognosis, and the effectiveness of therapeutic options.

**Methods:**

A total of 150 women were categorized into three groups: normal, benign mass, and BC. Participants were selected and evaluated at the cancer clinic; fasting blood samples were collected, and total antioxidant capacity (TAC), oxidized low-density lipoprotein (Ox-LDL), cancer antigen (CA) 15-3, and carcinoembryonic antigen (CEA) were measured. Subsequently, statistical analysis was performed to compare the levels of these parameters in different groups and examine the analytical performance of TAC and Ox-LDL in BC.

**Results:**

In patients with malignancy, the serum level of TAC was significantly decreased compared with the benign group (8.3 U/mL and 16.04 U/mL, respectively) (*P* < 0.001). Healthy controls exhibited higher levels of TAC (43.4 U/mL). The levels of Ox-LDL in BC were significantly increased in both malignant and benign groups (3,831 pg/mL and 1,234 pg/mL, respectively) compared with normal controls (682 pg/mL) (*P* < 0.001). CEA and CA15-3 were drastically increased in the BC groups compared with the control group. A significant area under the curve was observed in the receiver operating characteristic (ROC) curve analysis for TAC (0.975, *P* < 0.001) and Ox-LDL (0.986, *P* < 0.001).

**Conclusions:**

This study revealed that patients with BC had lower TAC and higher Ox-LDL serum levels, indicating elevated oxidative stress. These levels may serve as promising monitoring parameters in BC.

## Introduction

Breast cancer (BC) is a widespread and potentially fatal illness that affects millions of women globally [1]. Studies exploring the mechanisms underlying BC progression and treatment are crucial for developing effective therapies. One area of interest in BC research is evaluating the patients’ lipid peroxidation levels and total antioxidant status. Lipid peroxidation is a process that involves the oxidative modification of lipids, especially low-density lipoproteins (LDLs), caused by reactive oxygen species (ROS). This process can damage cells and tissues, leading to serious pathologies [2]. On the contrary, dietary antioxidants and/or antioxidant defense systems in the body play a vital role in protecting cells from oxidative stress [3, 4]. Understanding the balance between lipid peroxidation and antioxidant status in patients with BC might yield significant knowledge regarding disease progression, possible therapeutic approaches, and management options to enhance patient outcomes.

An assessment of lipid peroxidation and overall antioxidant status in patients diagnosed with BC has garnered considerable attention in the medical community as researchers strive to comprehend the underlying mechanisms of this disease in a better way. Kangari et al. [5] identified that patients with BC had markedly elevated levels of malondialdehyde (MDA) in the plasma, a marker for lipid peroxidation, in comparison with healthy individuals. The researchers concluded that this increase in plasma MDA levels is an important risk factor for BC and that the condition of oxidative stress was associated with the development and progression of BC. Didžiapetrienė et al. [6] investigated the oxidative stress biomarkers in patients with BC in preoperative and postoperative periods and reported the significance of these biomarkers in evaluating oxidative stress in patients with BC. Moreover, Delimaris et al. [7] observed the possible involvement of oxidized LDL (Ox-LDL) in the process of malignancy in 32 patients diagnosed with breast or ovarian cancer.

In addition to lipid peroxidation, a considerable body of research has explored the total antioxidant status in patients with BC. A study by Khalaf et al. [8] (2021) demonstrated that patients with BC had lower levels of antioxidants such as glutathione and ceruloplasmin compared with healthy individuals. Pathophysiological processes such as diabetes, degenerative diseases, atherosclerosis, and carcinogenesis have been linked to oxidative stress. It refers to an imbalance between the generation of oxidants and the antioxidant defense mechanisms [9–11]. Lipid peroxidation, a key consequence of oxidative stress, leads to the formation of reactive aldehydes and the oxidation of native LDLs, which can damage cellular structures and promote tumorigenesis. The imbalance between ROS production and antioxidant defense mechanisms may contribute to increased oxidative stress in patients with BC.

Evaluating lipid peroxidation and total antioxidant status in patients with BC holds immense clinical significance as it provides valuable insights into disease progression, patient prognosis, and treatment outcomes. Monitoring these biomarkers can aid healthcare providers in tailoring personalized therapeutic strategies to reduce oxidative stress, enhance antioxidant capacity, and improve patient outcomes. Additionally, targeting lipid peroxidation pathways or boosting antioxidant defenses may represent novel therapeutic approaches to manage BC and mitigate treatment-related side effects.

This study primarily aimed to analyze the levels of circulating biomarkers associated with oxidative stress comparatively. Specifically, it focused on assessing total antioxidant capacity (TAC) and Ox-LDL levels in patients diagnosed with BC and healthy controls. The intention was to determine the potential value of the quantitative analysis of these biomarkers and circulating markers, i.e., carcinoembryonic antigen (CEA) and cancer antigen (CA) 15-3, in diagnosing BC. Exploring the effect of lipid peroxidation and total antioxidant status on the advancement of BC provides opportunities for precision medicine and tailored treatment approaches.

## Materials and methods

### Participants

This study included 50 women diagnosed with benign breast mass (a mean age of 33.9 ± 1.9 years) and an equal number of women diagnosed with BC, mainly in the postmenopausal age group and not undergoing anticancer treatment (a mean age of 50.6 ± 1.5 years). This research involved selecting and examining patients at the cancer clinic of King Abdallah Medical City in Makkah, Saudi Arabia. The control group comprised 50 women volunteers (a mean age of 40.7 ± 0.93 years). All groups were matched in terms of age, weight, and menopausal status. Fasting blood samples were collected. The serum was separated by centrifuging the clotted samples at 3,500–4,000 rpm and stored at −20°C until analysis.

### Ethical approval

This study complied with the ethical standards specified in the 1975 Declaration of Helsinki, and clearance was obtained from the medical ethics committee of the College of Medicine at Umm Al-Qura University in Makkah, Kingdom of Saudi Arabia (Approval Number: HAPO-02-K012-2022-09-1183). Each participating patient and control provided written informed consent.

### Characteristics of the study groups

All participants underwent a clinical examination and completed a questionnaire that included aspects of their medical and family history. Individuals with positive neoadjuvant chemotherapy or a history of malignancy, radiotherapy, or/and chemotherapy were excluded. Blood samples were obtained before any surgical intervention.

The medical records of the participants involved in the study were examined to gather information from cytopathology reports, which contained details about the stage of the tumor, its features, and the status of ER and PR. Information regarding demographic features, including reproductive factors (such as age at menarche, age at menopause, age at first full-term pregnancy, number of full-term pregnancies, and prior use of external hormones such as hormone replacement therapy and oral contraceptives), medical history, and tobacco use, was collected via a thorough medical history and clinical examination. Data regarding cancer occurrence, including BC and other types, were collected from immediate (parents and siblings) and extended family members (grandparents, uncles, and aunts). BC was pathologically staged according to the TNM classification [12].

### Determination of serum levels of TAC

The serum levels of TAC were measured using a competitive inhibition enzyme immunoassay kit [MBS9304157, My BioSource, Sunny Southern California, San Diego (USA)] as per the given test protocol (https://www.mybiosource.com/).

### Determination of serum levels Ox-LDL

The serum levels of Ox-LDL were quantified using a competitive inhibition enzyme immunoassay kit (SEA527Hu 96 Testes, Cloud-Clone Corp, Houston, USA) according to a predetermined assay protocol (http://www.Cloud.Clone.US).

### Determination of serum levels of CA15-3

The serum levels of CA15-3 were measured using an ELISA kit provided by My BioSource, Inc., located in San Diego, USA. The CA15-3 ELISA test is a modified version of the solid-phase sequential sandwich ELISA. Biotinylated monoclonal antibodies and samples are introduced into wells coated with streptavidin. The CA15-3 in the patient sample forms a complex with the capture antibody modified with biotin. Simultaneously, the biotinylated antibody binds to the streptavidin-coated plate. Following the wash stage, the anti-CA15-3-horseradish peroxidase (HRP) enzyme conjugate is introduced and binds to the trapped CA15-3, creating a sandwich structure. The unbound antibodies are rinsed away. Upon adding the TMB substrate, a blue color is produced. The level of CA15-3 is directly correlated with the intensity of the color. A standard curve is plotted to establish the relationship between the intensity of the color and the level of CA15-3.

### Determination of serum levels of CEA

The serum level of CEA was quantified using an ELISA kit manufactured by Cloud-clone Corp. and assembled by US Co Life Science Inc. USA. A sandwich enzyme immunoassay kit is presented here. A precoated antibody specifically targeting the CEA has been applied to the microtiter plate included in this kit. In the next step, standards or samples are added to the appropriate wells of a microtiter plate together with a biotin-conjugated antibody that selectively binds to the CEA. Furthermore, the avidin-HRP conjugate is added to each microplate well and incubated. Upon introducing the TMB substrate solution, only the wells containing CEA, biotin-conjugated antibody, and enzyme-conjugated avidin would exhibit a noticeable color change. The enzyme-substrate interaction is stopped by adding sulfuric acid, and the subsequent color change is measured quantitatively using a spectrophotometer at 450 ± 10 nm. The CEA levels in the sample are measured by comparing the optical density of the samples with the standard curve. The measured value varied between 19.6 ng/mL and 1,250 ng/mL.

### Statistical analysis

The data were analyzed using the SPSS (version 20, Sydney, NSW, Australia) software. Quantitative data were reported as the mean values ± standard error (SE), whereas qualitative data were presented as frequencies and percentages. Statistical analysis was performed using an independent sample *t*-test for parametric variables to compare between two groups. One-way analysis of variance with post-hoc multiple comparisons test was used to evaluate the mean difference among the malignant, benign, and healthy control groups. Pearson’s correlation coefficient and linear regression analysis were performed to analyze the correlation between oxidative stress parameters (TAC and Ox-LDL) and tumor markers (CEA and CA15-3). The Kolmogorov-Smirnov test and normal plot were used to determine the normality of the data. To assess the diagnostic accuracy of the serum TAC, Ox-LDL, and CA15-3 levels, the receiver operating characteristic (ROC) curve analysis was performed on the dataset. The measurement accuracy was determined by calculating the area under the ROC curve, commonly referred to as AUC. A test with an area of 1 is considered ideal, whereas a test with an area of 0.5 is considered completely ineffective. The accuracy of a diagnostic test may be classified using a standard academic point system. An accuracy score of 0.9–1 is considered outstanding (A), 0.8–0.9 is acceptable (B), 0.7–0.8 is fair (C), 0.6–0.7 is bad (D), and 0.5–0.6 is failed (F). For each statistical analysis, *P*-values < 0.05 were deemed significant.

## Results

### Characteristics of the study groups

Pretreatment blood samples were acquired from patients with BC diagnosed with the disease. The diagnosis was verified using histological and clinical data and medical records. Table 1 summarizes the clinical features and demographic data of patients with BC and those with benign conditions. Patients with BC and benign conditions were matched in terms of age with the control group. In a sample of 50 patients with BC, 6 (12%) patients were classified as grade I, 31 (62%) as grade II, 11 (22%) as grade III, and 2 (4%) as grade IV (Table 1). Immunohistochemical data showed that 70% of the samples were estrogen-receptor-positive (ER^+^), 56% were progesterone-receptor-positive (PR^+^), and 28% were human epidermal growth factor receptor 2 positive (Her2^+^) (Table 1). Of the 50 patients with BC, 48 (96%) presented with a mass, whereas 2 (4%) did not have a mass. In addition, 2 (4%) experienced discharge, which included blood, whereas 48 (96%) did not have any discharge. Among a group of 50 patients with benign breast masses, 39 were identified as having fibroadenoma. In contrast, the remaining 11 patients were diagnosed with various other forms of benign breast conditions, such as granulomatous mastitis, papilloma, fibroglandular tissue, and ductal ectasia (Table 1). Histopathological examinations of 50 patients with BC revealed that 47 (94%) had invasive ductal carcinoma, whereas 3 (6%) had lobular carcinoma. Of the patients with BC, 37 (74%) had lymph node involvement, whereas 13 (26%) did not have lymph node involvement. Moreover, 21 (42%) had cancer metastasis, whereas 29 (58%) did not exhibit metastasis, as indicated in Table 1.

**Table 1:** The clinical and characteristic features of the studied groups

**Characteristic features/clinicopathological parameters**	**Malignant (*N* = 50)**			**Benign (*N* = 50)**			**Control (*N* = 50)**			***P* value**
	**No**	**(%)**	**Mean ± SE**	**No**	**(%)**	**Mean ± SE**	**No**	**(%)**	**Mean ± SE**	
**Age**										
< 40	8	16%	50.6 ± 1.5	33	66%	33.9 ± 1.9	21	42%	40.8 ± 0.93	0.076
40–60	34	68%		15	30%		29	58%		
> 60	8	16%		2	4%		0	0		
**Menstrual phase (in present)**										
Premenopausal	29	58%		48	96%		33	66%		
Postmenopausal	21	42%		2	4%		17	34%		
**Marital status**										
Married	34	68%		29	58%		32	64%		
Single	10	20%		20	40%		17	34%		
Divorced	3	6%		1	2%		1	2%		
Widowed	3	6%		0	0		0	0%		
**Lactation history**										
Lactating	34	68%		27	54%		24	48%		
Non-lactating	16	32%		23	46%		26	52%		
**Family history of BC**										
Yes	7	14%		6	12%		5	10%		
No	43	86%		44	88%		45	90%		
**Benign types**										
Fibro adenoma				39	78%					
Others				11	22%					
**Side of complained**										
Right breast	22	44%		27	54%					
Left breast	28	56%		17	34%					
Both sides	0	0		6	12%					
**Cancer types**										
Invasive ductal carcinoma	47	94%								
Lobular carcinoma	3	6%								
**Cancer metastasis**										
Yes	21	42%								
No	29	58%								
**LN involvement**										
Yes	37	74%								
No	13	26%								
**Grade**										
Grade I	6	12%								
Grade II	31	62%								
Grade III	11	22%								
Grade IV	2	4%								
**Mass**										
Yes	48	96%		41	82%					
No	2	4%		9	18%					
**Discharge**										
Yes	2	4%		4	8%					
No	48	96%		46	92%					
**Immunohistochemistry (IHC)**										
Estrogen receptors (ER)	35	70%								
Progesterone receptors (PR)	28	56%								
Human epidermal growth factor receptor-2 (Her2)	14	28%								

BC: breast cancer; SE: standard error

### Serum levels of TAC, Ox-LDL, CA15-3, and CEA

**Figure 1:**
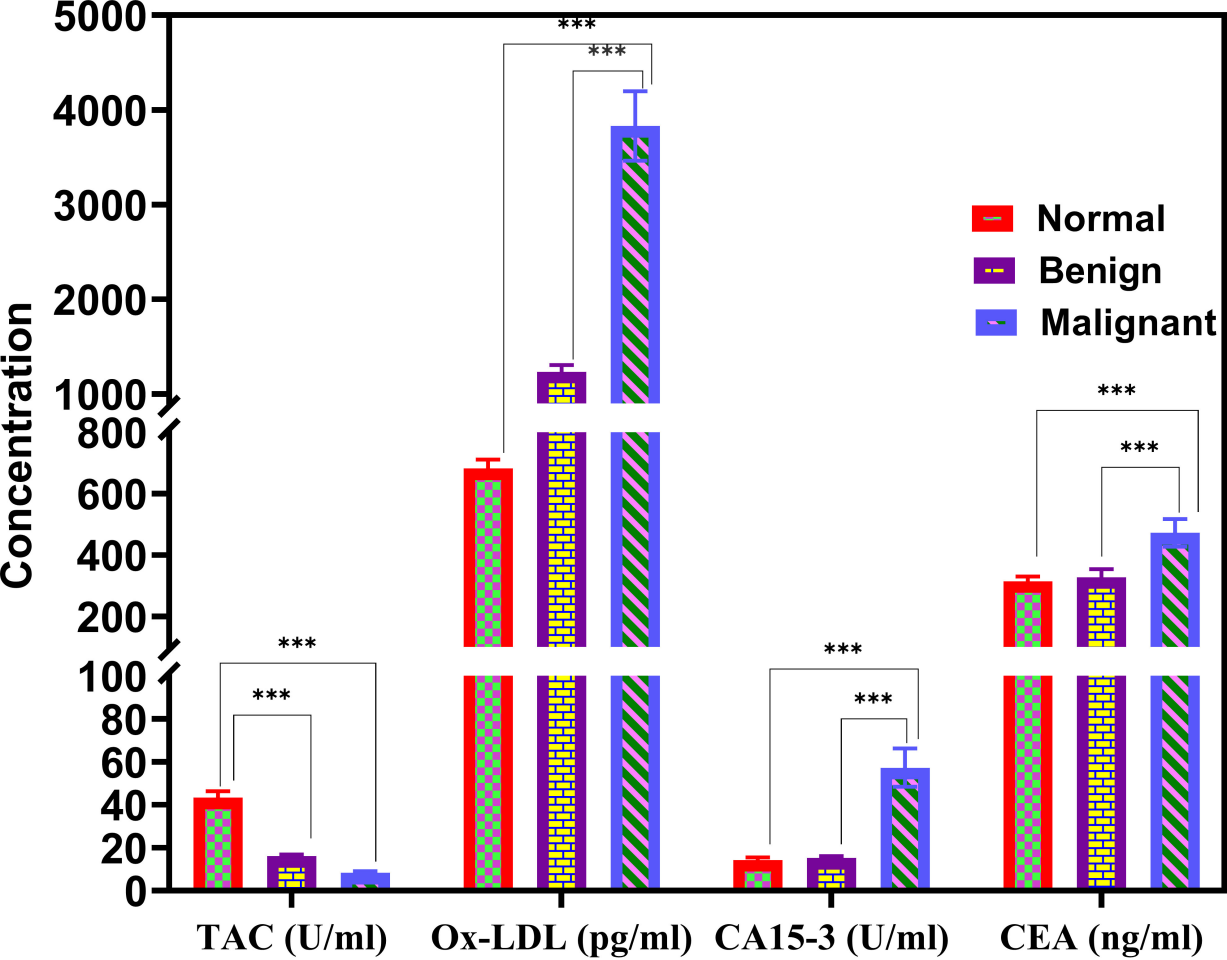
**Comparison of serum levels of TAC, Ox-LDL, CA15-3, and CEA among the benign, BC, and control groups**. ^***^ *P* < 0.001. TAC: total antioxidant capacity; Ox-LDL: oxidized low-density lipoprotein; CA: cancer antigen; CEA: carcinoembryonic antigen; BC: breast cancer

The serum level of TAC was highly and significantly decreased in patients with BC and benign lesions, with mean values of 8.3 U/mL and 16.04 U/mL, respectively, as shown in Figure 1, in comparison with normal controls (43.4 U/mL) (*P* < 0.001).

Moreover, the serum level of Ox-LDL was highly and significantly increased in patients with BC compared with the benign disease group, with mean values of 3,831 pg/mL and 1,234 pg/mL (*P* < 0.001), respectively (Figure 1). Furthermore, patients with BC displayed significantly higher means of Ox-LDL in comparison with normal healthy controls (682 pg/mL) (*P* < 0.001).

Also, the BC group demonstrated a significant increase in the mean values of the other two parameters, i.e., CEA and CA15-3, compared with the control group. The mean CEA levels were found to be 472.56, 328.42, and 314.55 ng/dL in the BC, benign, and control groups, respectively (*P* < 0.001). In contrast, the means of CA15-3 were 57.28, 15.16, and 14.35 U/mL in the BC, benign, and control groups, respectively, with a significant difference (*P* < 0.001), as shown in Figure 1.

### Correlations of TAC, Ox-LDL, and CA15-3

**Figure 2:**
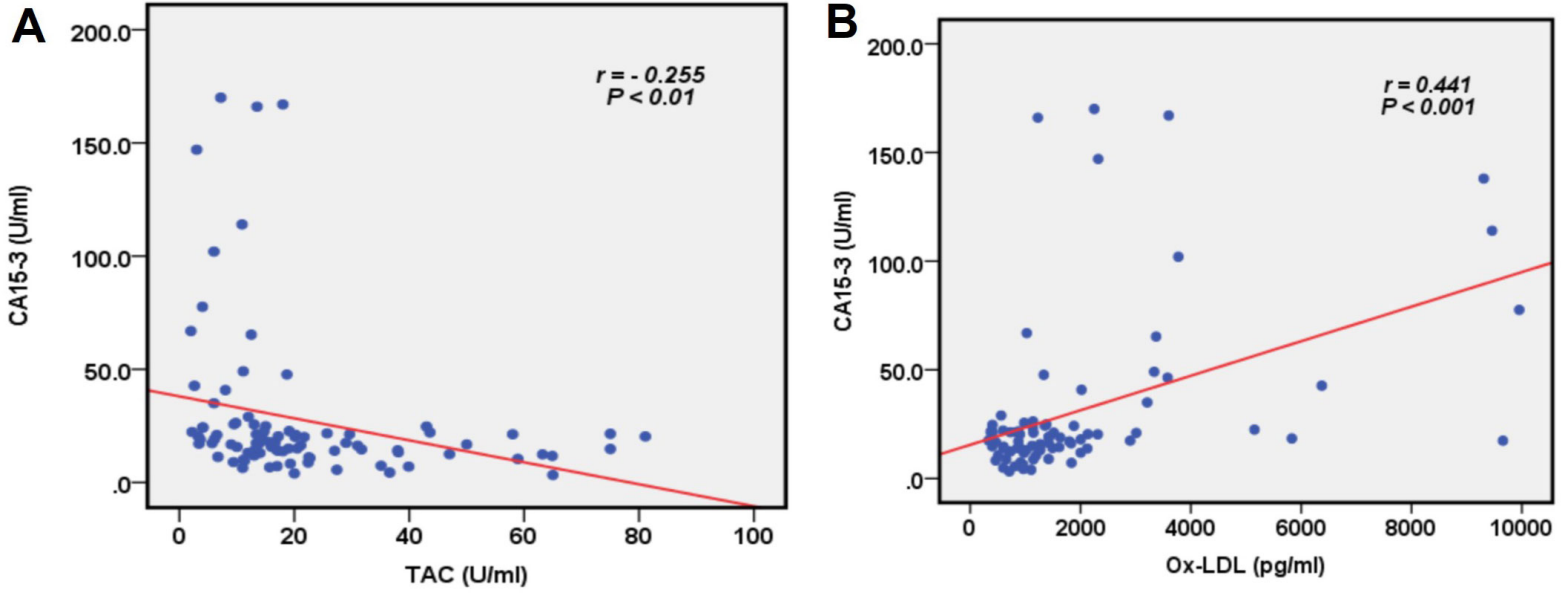
**Correlation between the serum levels of TAC, Ox-LDL, and CA15-3**. (**A**) TAC and CA15-3; and (**B**) Ox-LDL and CA15-3. CA: cancer antigen; TAC: total antioxidant capacity; Ox-LDL: oxidized low-density lipoprotein

The studied marker TAC showed a weak negative correlation with CA15-3 (*r* = −0.255, *P* < 0.01). A significant positive correlation between Ox-LDL and CA15-3 was observed (*r* = 0.441, *P* < 0.001), as shown in Figure 2.

### Association between predictive immunohistochemistry and TAC and Ox-LDL

**Figure 3:**
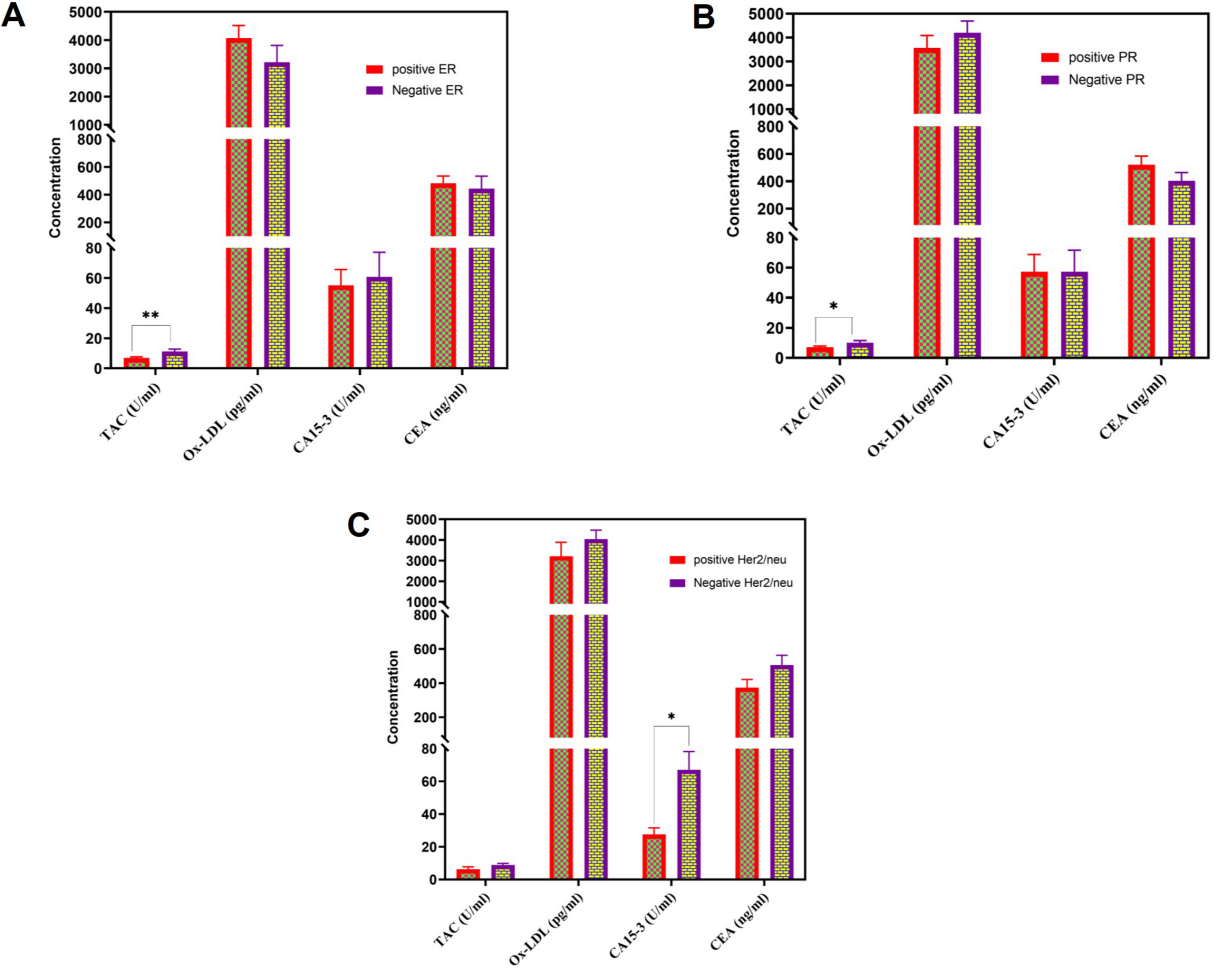
**The serum levels of TAC, Ox-LDL, CA15-3, and CEA in patients with BC who had distinct histopathological observations**. These observations included (**A**) positive estrogen receptors (ER); (**B**) positive progesterone receptors (PR); and (**C**) positive human epidermal growth factor receptor-2 (Her2/neu). TAC: total antioxidant capacity; Ox-LDL: oxidized low-density lipoprotein; CA: cancer antigen; CEA: carcinoembryonic antigen; BC: breast cancer

Figure 3 displays the variations in TAC, Ox-LDL, CA15-3, and CEA levels among patients with BC who had specific histopathological characteristics.

### Diagnostic performance of serum TAC and Ox-LDL for BC

**Figure 4:**
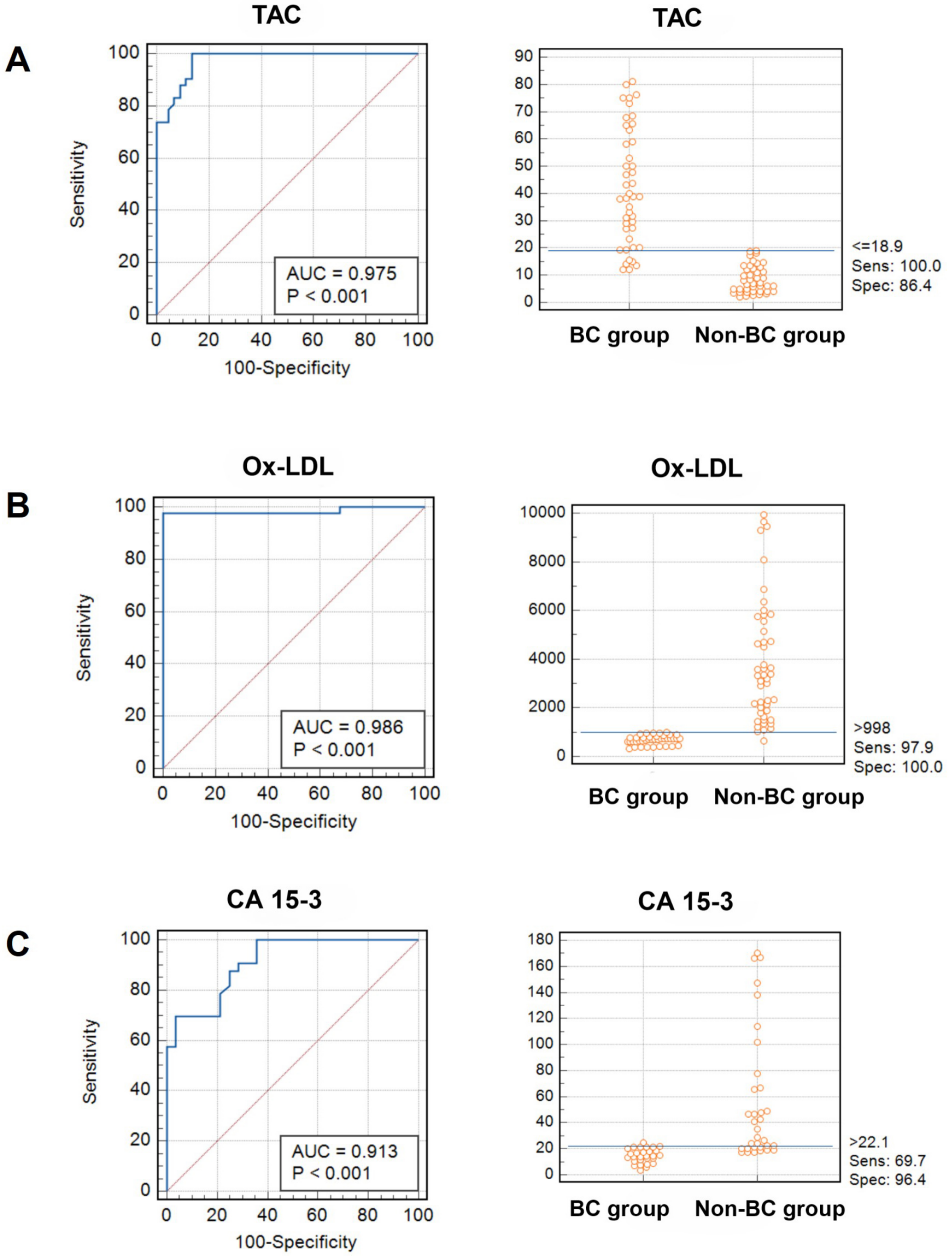
**Analysis of ROC curves and interactive dot diagrams of parameters investigated in patients with BC**. Sens represents sensitivity, spec denotes specificity, and blue lines signify the designated cutoff value for each parameter; BC: breast cancer; ROC: receiver operating characteristic

The ROC curve was examined to assess the ability of the serum levels of TAC to discriminate between samples with and without BC. Figure 4 depicts the AUC. The data analysis of the ROC curve revealed a significant AUC with a value of 0.975 and a statistically significant *P*-value of < 0.001. The sensitivity and specificity were determined to be 100% and 86.4%, respectively, using a cutoff value of TAC equal to 18.9 U/mL (Table 2).

**Table 2:** Diagnostic data of serum levels of TAC, Ox-LDL, and CA15-3 using ROC curve

**Variable**	**AUC**	**SE**	**95% CI**	**Sensitivity**	**Specificity**	**Cutoff value**
TAC	0.975	0.0247	0.870 to 0.997	100	86.4	18.9
Ox-LDL	0.986	0.0225	0.887 to 0.999	97.9	100	998
CA15-3	0.913	0.0518	0.736 to 0.947	69.7	96.4	22.1

SE: standard error; TAC: total antioxidant capacity; Ox-LDL: oxidized low-density lipoprotein; CA: cancer antigen; ROC: receiver operating characteristic

In addition, the ROC curve analysis was performed to evaluate the discriminatory ability of the Ox-LDL test in distinguishing between samples with and without BC. Figure 4 displays the AUC.

The data analysis of the ROC curve revealed a significant AUC with a value of 0.986, indicating statistical significance (*P* < 0.001). In Table 2, the sensitivity and specificity were determined to be 97.9% and 100%, respectively, using a cutoff value of 998 pg/mL for Ox-LDL.

## Discussion

Cancer is one of the leading causes of mortality globally, with BC being the predominant malignancy in women, representing 30% of all cancers diagnosed in women annually [13]. Biomarkers that provide information on disease progression are used as diagnostic tools and attract considerable attention. A limited number of markers are employed to enhance our comprehension of the role of oxidative stress in cancer pathophysiometry [14]. This study assessed the levels of biomarkers associated with oxidative stress in patients with BC and healthy individuals. Specifically, it focused on TAC and Ox-LDL and the potential diagnostic value of CEA and CA15-3 in BC. In previous studies, lipid peroxidation and TAC have shown an intricate association in patients with BC. Investigations have revealed that lipid peroxidation is substantially elevated in patients with BC, suggesting increased oxidative stress [15, 16]. Furthermore, the oxidative/antioxidant profile in patients with BC has been reported to be influenced by various prognostic factors, such as cancer stage, tumor size, and molecular markers. This finding highlights the dynamic interplay between oxidative stress and antioxidant capacity in BC progression [14].

Our study’s findings confirm prior research indicating that excessive ROS production or insufficient elimination might contribute to cancer progression. The results of TAC and Ox-LDL levels support this finding [6, 14].

Ox-LDL levels were increased, and TAC levels were decreased in patients with BC compared to those in the control group. Moreover, the highest levels of Ox-LDL were observed in patients with malignancy, as shown in Figure 1. These findings of decreased antioxidant defense, as inferred from low levels of TAC in patients with malignancy, suggest that oxidative stress is strongly correlated with disease development. These outcomes agree with those from previous studies, implying that disturbance in the antioxidant defense and oxidative stress promote DNA damage in cancers and may be linked to benign and malignant tumors [17, 18].

Tests based on multiple markers can considerably enhance the ability to detect heterogeneous tumor cells compared with single marker assays [19]. While other serum-based tumor markers have been identified for BC, the most commonly used are CA15-3 and CEA [20]. BC is the only factor that can lead to elevated levels of CA15-3. Combining the preoperative level of CA15-3 with current prognostic markers can help predict outcomes in patients who have just been diagnosed with BC. A study has proposed improving tumor cell identification sensitivity by evaluating numerous tumor indicators in a single blood sample [21].

The TAC values, as indicated by our data (with a sensitivity of 100% and a specificity of 86.4%) and depicted in Figure 4, have the potential to serve as a marker for distinguishing between patients with BC and healthy individuals. This value can potentially serve as a screening signal for the early detection of BC. Additionally, if the treatment goal is to address oxidative stress, it might be utilized as an indicator of illness to monitor and assess the effectiveness of the therapy. Prior research has observed that antioxidant defenses can be enhanced by physiological signals, dietary components, or possibly pharmacological intervention [9, 14]. In our study, the diagnostic value of Ox-LDL exhibited considerably good performance, especially in excluding negative cases of BC, as reflected by its high specificity of 100% sensitivity of 97.9%, and AUC of 0.986.

Patients with BC with negative estrogen receptors (ER) and progesterone receptors (PR) had considerably elevated levels of TAC, whereas those with BC with negative Her2/neu showed significantly higher levels of CA15-3. Figure 3 demonstrates the absence of significant differences in the values of these parameters between patients with BC who tested positive for ER and PR. Multiple studies have established a correlation between BC triggered by estrogen and the presence of oxidative stress [22]. Furthermore, oxidative DNA damage has been reported to be highly correlated with the presence of ER and is higher in BC issues than in normal breast tissues [23, 24]. These findings suggest that oxidative stress might be related to ER expression and that further investigations are needed to explore this relationship.

A significant negative correlation was noted between TAC and the biomarkers under investigation, i.e., CA15-3 and CEA, which supports the proposed relationship with BC (Figure 2A). Furthermore, Ox-LDL levels were significantly and positively correlated with the two investigated biomarkers, as illustrated in Figure 2B. This observation signifies that the pattern of Ox-LDL level increases with both known biomarkers in an almost comparable manner in malignant, benign, and normal samples. Our findings could potentially endorse the diagnostic utility of TAC and Ox-LDL in BC. To the best of our knowledge, previous studies evaluating the state of oxidative stress in patients with BC have focused on one or a few oxidants/antioxidant indicators. The use of these biomarkers in clinical settings is still not well-established [25, 26]. Although these results show a correlation between the investigated biomarkers and the onset and development of BC, they are insufficient to indicate the patients’ actual oxidative stress state or to identify an appropriate panel of diagnostic or prognostic biomarkers for BC. Using Ox-LDL, TAC, and oxidative stress in managing BC holds immense potential as an approach that can be further developed to enhance patient outcomes and improve overall management. However, these biomarkers should be interpreted cautiously and based on proper assessments of analytical and clinical validations. The reason is that they are highly correlated to many other pathologic scenarios, such as hyperglycemia [2], cancers [14, 17], aging [27], and cardiovascular events [28]. Based on the solid foundations of these established relationships and the relatively small sample size in this study, further research is required to validate the application of these biomarkers in clinical settings.

### Conclusion

This study observed that patients with BC exhibited lower TAC levels and higher Ox-LDL levels in the serum, indicating elevated oxidative stress compared with the control group. This finding suggests a potential association between increased oxidative stress and BC. Based on our observations, TAC and Ox-LDL levels could be additional parameters for monitoring cancer prevention, diagnosis, and treatment. However, the exact mechanism behind the elevated oxidative stress, whether it stems from increased ROS production or reduced antioxidant defenses, is yet to be elucidated. Therefore, further studies on oxidative stress and antioxidant therapy in BC are recommended.

## Abbreviations

BC: breast cancer
CA: cancer antigen
CEA: carcinoembryonic antigen
ER: estrogen receptors
Her2: human epidermal growth factor receptor-2
LDLs: low-density lipoproteins
Ox-LDL: oxidized low-density lipoprotein
PR: progesterone receptors
ROC: receiver operating characteristic
ROS: reactive oxygen species
SE: standard error
TAC: total antioxidant capacity

## Declarations

### Availability of data and materials

The raw data supporting the conclusions of this manuscript will be made available by the authors, without undue reservation, to any qualified researcher.

### Copyright

© The Author(s) 2025.
